# Challenging perspectives on the cellular origins of lymphoma

**DOI:** 10.1098/rsob.160232

**Published:** 2016-09-28

**Authors:** Tim I. M. Malcolm, Daniel J. Hodson, Elizabeth A. Macintyre, Suzanne D. Turner

**Affiliations:** 1 Division of Molecular Histopathology, Department of Pathology, University of Cambridge, Lab Block Level 3, Box 231, Addenbrooke's Hospital, Cambridge CB2 0QQ, UK; 2 Department of Haematology, University of Cambridge, Cambridge, UK; 3 Hematology and INSERM1151, Institut Necker-Enfants Malades, Université Sorbonne Paris Cité at Descartes and Assistance Publique-Hôpitaux de Paris, Paris 75743 Cedex 15, France

**Keywords:** lymphomagenesis, T cell, anaplastic large cell lymphoma

## Abstract

Both B and T lymphocytes have signature traits that set them apart from other cell types. They actively and repeatedly rearrange their DNA in order to produce a unique and functional antigen receptor, they have potential for massive clonal expansion upon encountering antigen via this receptor or its precursor, and they have the capacity to be extremely long lived as ‘memory’ cells. All three of these traits are fundamental to their ability to function as the adaptive immune response to infectious agents, but concurrently render these cells vulnerable to transformation. Thus, it is classically considered that lymphomas arise at a relatively late stage in a lymphocyte's development during the process of modifying diversity within antigen receptors, and when the cell is capable of responding to stimulus via its receptor. Attempts to understand the aetiology of lymphoma have reinforced this notion, as the most notable advances to date have shown chronic stimulation of the antigen receptor by infectious agents or self-antigens to be key drivers of these diseases. Despite this, there is still uncertainty about the cell of origin in some lymphomas, and increasing evidence that a subset arises in a more immature cell. Specifically, a recent study indicates that T-cell lymphoma, in particular nucleophosmin-anaplastic lymphoma kinase-driven anaplastic large cell lymphoma, may originate in T-cell progenitors in the thymus.

## 1. Lymphoma as a cancer of the immune system

Lymphomas belong to a family of tumours that affect haematopoietic and lymphoid tissues, which includes leukaemia. Broadly, lymphoma is distinguished from leukaemia in its clinical presentation, where lymphoma predominately involves lymph nodes, and leukaemia the blood and bone marrow, and by the fact that lymphomas arise from cells that reside in the lymphatic system rather than in the bone marrow. However, in the context of the cell of origin this is perhaps a rather nebulous distinction, and there is evidence that chronic lymphocytic leukaemia (CLL) may emerge in differentiated/mature lymphocytes in the periphery. For example, it has been suggested that CLL may arise in marginal-zone B cells [1], regulatory B cells [2] or memory B cells [3], with B-cell receptor (BCR) signalling representing a key promoting factor [1]. Similarly, in adult T-cell leukaemia/lymphoma (ATLL), a mature T-cell neoplasm, the cell of origin is a post-thymic lymphocyte infected by the human T-cell lymphotropic virus (HTLV-1). Notably, it presents as a leukaemia in more than two-thirds of patients, and as a lymphoma in the remainder [4]. At the other extreme, B/T-lymphoblastic leukaemia/lymphoma is a neoplasm of immature B cells or T cells, arising from precursor B cells in the bone marrow or precursor T cells in the thymus [5]. These are arbitrarily considered as two diseases, acute lymphoblastic leukaemia (ALL) and lymphoblastic lymphoma (LBL), based on the presence of more or less than 25% bone marrow blasts, despite the fact that they share many morphological and immunophenotypic features [6]. Thus, it is quite likely that in terms of the mechanisms of tumourigenesis and the cell types involved, the aetiology of lymphoma and leukaemia overlap. However, for the sake of clarity and brevity, this review is only considering the origin of cancer in lymphocytes that leads to a disease that has been classified as lymphoma.

Considering lymphomas specifically, the progression of the disease has yet to be completely understood. At some point along the journey of a lymphocyte from its haematopoietic stem cell origin to its fully differentiated mature destination, a build-up of genetic mutations can lead to the transformation and subsequent clonal expansion of this lymphocyte, resulting in the formation of lymphoma. A key initiating genetic event is in some cases a chromosomal translocation, usually resulting from a balanced reciprocal recombination event. Illustrating this, chromosomal translocations are present in up to 90% of non-Hodgkin lymphoma (NHL), the majority lymphoma category [7]. Lymphomas, like most cancer types, are extremely diverse, and a number of different entities have been described, distinguished from each other by morphologic, immunophenotypic and genetic features [8]. Attempts to understand lymphoma aetiology as a whole has been challenging in the face of such apparent complexity. It is likely that in order to gain a deeper understanding of this disease's origins and development, it is better to regard it as a mixture of different disease entities with potentially varying aetiologies. This review discusses the cellular origin of lymphoma, specifically with regard to the state at which differentiation arrest occurs in order to give rise to the plethora of NHL sub-types observed.

## 2. Development of lymphocytes

**Figure 1.**
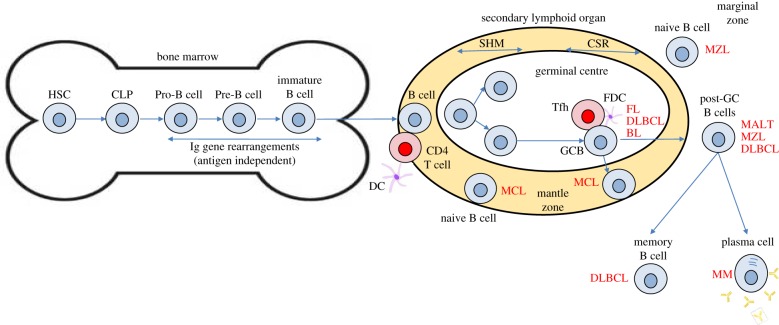
B-cell development and the origins of B-cell lymphoma. B-cell development commences in the bone marrow whereby committed B cells rearrange the immunoglobulin (Ig) genes to generate a B-cell receptor. On entering the periphery, B cells congregate in lymphoid tissue wherein antigen-dependent B-cell development takes place. Naive B cells on encountering antigen become activated and generate germinal centres in which the processes of somatic hypermutation (SHM) and class switch recombination (CSR) take place in the dark and light zones, respectively. Studies of the status of the Ig genetic regions within lymphoma cells can align their origin in either pre- or post-germinal centre B cells. For example, the majority of mantle cell lymphoma (MCL) do not display mutations within V-region genes suggesting they have not passed through the germinal centre and are therefore presumed naive. The origin of lymphomas is also assumed from their growth pattern and cell surface expression of proteins indicative of stages of B-cell development. MALT, mucosal-associated lymphoid tissue lymphoma; MZL, marginal-zone lymphoma; DC, dendritic cell; DLBCL, diffuse large B-cell lymphoma; CLP, common lymphoid progenitor; HSC, haemopoietic stem cell; GC, germinal centre B cell; FDC, follicular dendritic cell; Tfh, follicular helper T cell; FL, follicular lymphoma; BL, Burkitts lymphoma. Acronyms in red denote the presumed cell of origin of the indicated lymphoma sub-type.

**Figure 2.**
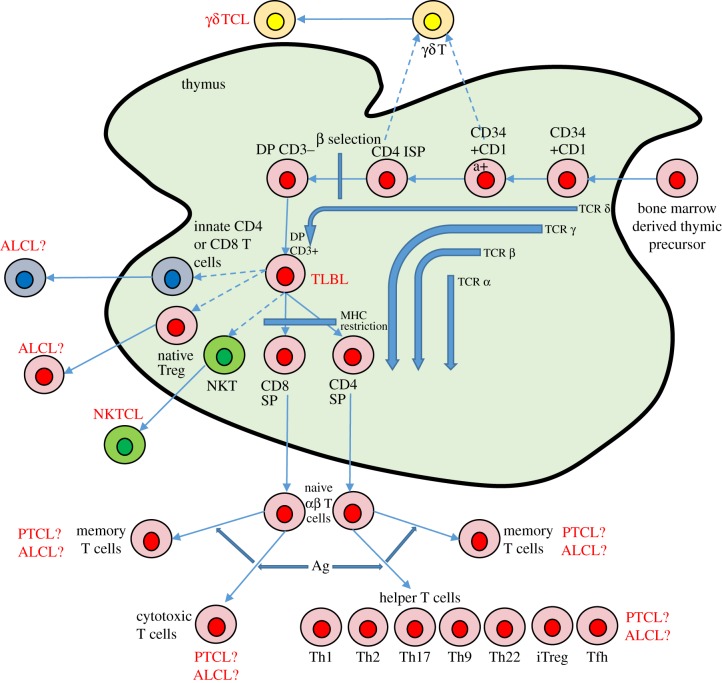
Human T-cell development and the origins of T-cell lymphoma. T-cell development occurs in the thymus wherein early thymic progenitors from the bone marrow pass through the double negative (DN) stages rearranging the T-cell receptor genes in an ordered process to enable bypass of the beta selection checkpoint or differentiation into γδ T cells. Following both positive and negative selection, surviving T cells differentiate into either innate, regulatory (Treg), natural killer/T (NKT), cytotoxic or helper (Th) T-cell subsets. In an antigen-dependent process, these cells can further differentiate into distinct T-cell subsets or memory cells. T-lymphoblastic lymphoma (TLBL) arises from the immature thymic T cells whereas peripheral T-cell lymphoma (PTCL) has origins in mature, terminally differentiated T cells. By contrast, the origins of anaplastic large-cell lymphoma (ALCL), a peripheral T-cell disease, are controversial. Ag, antigen; TCL, T cell lymphoma; NKTCL, natural killer T-cell lymphoma. Dashed arrows represent less well-established differentiation pathways.

Defining what makes a lymphocyte immature or mature is critical if we are to differentiate the origin of lymphomas on that basis. In simple terms, when a lymphocyte is no longer capable of giving rise to more than one haematopoietic lineage, and has assembled all the necessary structures to perform its function, it has reached maturity. For B cells, their period of immaturity spans their life in the bone marrow, broadly defined by the lack of cell surface immunoglobulin but with the presence of the cell surface protein CD19 (figure 1). The earliest B-cell precursors, pre-pro-B cells, can still give rise to T cells, natural killer (NK) cells and dendritic cells (DCs), but as they reach the pro-B-cell stage they lose multi-potentiality and begin assembling immunoglobulin, maturing further into the pre-B-cell and immature B-cell stages (accompanied by the expression of surface IgM). B cells complete their maturation in secondary lymphoid organs such as the spleen [9]. For T cells, their development begins in the thymus as their earliest precursor, the pro-T cell (which is CD34^+^CD1a^−^ in humans, CD4^−^ CD8^−^CD25^−^CD44^+^ (DN1) in mice), when they still have the potential to differentiate into the B-cell, myeloid, NK and DC lineages (figure 2 and reviewed in [10]). The precursor thymocyte is only committed to the T-cell lineage following rearrangement of T-cell receptor (TCR) δ, γ and then β regions at the early double positive (DP) stage (CD3^−^CD4^+^CD8^+^ in humans, CD4^−^CD8^−^CD25^+^CD44^−^ (DN3) in mice) [11]. Cells with productive TCRβ rearrangements give rise to αβ-T CD3^+^CD4^+^CD8^+^ DP cells, and complete their maturation in the thymus, becoming either CD4^+^ or CD8^+^ single positive (SP) cells following MHC restriction, before eventually migrating to the periphery [12] (figure 2).

Once they have reached maturity B lymphocytes undergo a series of processes that enable diversity in antigen recognition (somatic hypermutation and class switch recombination) within germinal centres of lymphoid follicles in secondary lymphoid organs (figure 1). Naive B cells present in the mantle zones of follicles on interaction with antigen presenting helper T cells are stimulated to move into the germinal centre whereupon they undergo the processes of somatic hypermutation and class switch recombination to emerge as activated post-germinal centre B cells [13]. Those B cells that express an antigen receptor with increased affinity for antigen (usually presented to the cells on the surface of follicular DCs) are then selected, the remaining cells undergoing apoptosis [14]. Mature B cells then exit into the periphery as antibody-producing plasma cells or long-lived memory cells [15].

## 3. Mechanisms of lymphomagenesis in ‘mature’ lymphocytes

### 3.1. Lymphoid cell developmental processes contribute to lymphomagenesis

Key to lymphomagenesis in mature lymphocytes is that most of the developmental processes described above have the potential to introduce oncogenic mutations. The formation of the antigen receptor in B and T cells involves recombination activating gene (RAG) 1/2 endonucleases initiating V(D)J recombination, leading to immunoglobulin or T cell receptor (TCR) assembly, respectively [16]. Further, V(D)J rearrangement may occur in mature B cells (receptor revision) [14] and may lead to translocation of the Ig loci to oncogenes including c-myc [15]. Class switch recombination, where the constant regions of the B cell receptor are changed, allows the effector function of the antibody to be altered [17]. Thus, when breakpoints that lead to oncogenic translocations are located in the switch regions of the heavy chain constant genes, it is probably a result of mistaken class switch recombination. This is the case for several translocations, including *MYC* in Burkitt lymphoma [18] and *BCL6* in diffuse-large-B cell lymphoma [19].

Somatic hypermutation in B cells generates ‘mutations’ within the immunoglobulin variable regions in a process largely mediated by activation induced cytidine deaminase (AID). This occurs during the B cell response to T-cell-dependent antigens, allowing B cells to be selected on the basis of improved affinity for the antigen [20]. However, this process can be a cause of malignancy, directly or indirectly. Directly, because it is capable of causing deletions or insertions that can lead to oncogenic translocations, such as MYC translocations in Burkitt lymphoma [21]. Indirectly, as by changing the affinity for antigen, somatic hypermutation may allow a previously ‘normal’ B cell to make a hyperactive response that could generate a malignancy as discussed below [22].

The causes of chromosomal translocations and other mutations in T cell lymphoma are less well understood and few have been described. The most well known is the anaplastic large-cell lymphoma (ALCL)-associated nucleophosmin-anaplastic lymphoma kinase (NPM-ALK), the consequence of a t(2;5)(p23;q35) event described further below and for which the responsible mechanism remains to be determined [23]. As such, data for mechanisms of mature T-cell lymphomagenesis are lacking in comparison with B-cell lymphomas, largely due to the relative scarcity of known driver mutations through which to investigate these diseases, their heterogeneity and their rarity. As such, some of the evidence for T-cell lymphomas initiating in mature T cells comes from serendipitous findings in mouse models. For example, deletion of the SWI/SNF-related regulator of gene expression SNF5 in mice leads to rapid onset of mature peripheral T-cell lymphoma (PTCL) [24]. In a model where expression of Snf5 was deleted in mature T cells but not at earlier stages of thymic development, it was shown that cells with a CD44^hi^CD122^lo^CD8^+^ memory-like phenotype accumulated, with the mice eventually developing CD8^+^ mature PTCL in the spleen, liver and lymph nodes [25]. However, snf5 deletion has not been reported in human PTCL (the region in which snf5 resides is deleted in 50% of prolymphocytic leukaemia [26]). However, these data do indicate that memory cells might be the source of T-cell lymphomagenesis, cells that inherently have the ability to self-renew and are long-lived enabling the acquisition of tumour-promoting mutations. Perhaps more relevant to human PTCL is the oncogenic driver, interleukin-2 inducible T-cell kinase-spleen tyrosine kinase (Itk-Syk) fusion protein which has been associated with a small number of cases of follicular-type PTCL and AITL [27,28]. Expression of Itk-Syk via CD4 promoter-driven Cre in transgenic mice leads to peripheral CD4 and/or CD8 SP T-cell lymphoma in mice with tumour cells having an activated T-cell phenotype (CD62^lo^CD44^hi^; also indicative of an effector memory T cell) [29]. Likewise, expression of lin28b, in this case from the haemopoeitic-ubiquitous vav promoter, leads to a PTCL-like disease in mice, although links to human disease are tenuous, with Lin28b reported as being overexpressed by on average 7.5-fold in PTCL, NOS (*n* = 50) [30]. In this latter case, tumour cells resemble follicular T cells, suggesting an origin in this mature cellular compartment.

### 3.2. Chronic antigenic stimulus and lymphomagenesis

#### 3.2.1. Bacteria and lymphoma

As the interaction of antigen with its antigen receptor on a lymphocyte leads to massive proliferation, it has long been supposed that exposure (perhaps chronic) to infection is an important factor in the formation of lymphoid cancers, and this idea has been strengthened further by recent studies of follicular lymphoma (FL). FL cells express Ig unusually marked by the presence of glycan chains terminating in mannose (as a result of somatic hypermutation-induced mutations of the Ig) which recognize lectin on presenting cells in the germinal centre (reviewed in [22]). Furthermore, the Igs of the lymphoma cells recognize lectins from opportunistic pathogens such as *Pseudomonas aeroginosa* and *Burkholderia cenocepacia*, suggesting a role for bacterial infection in the pathogenesis of this disease [31]. The association of bacterial infection with lymphomagenesis is not a new concept as the relationship of the bacterial species *Helicobacter pylori* with mucosal-associated lymphoid tissue (MALT) lymphoma is well documented, although the mechanism in this scenario is not a direct interaction as described below [32].

MALT lymphoma is a B-cell lymphoma that is associated with bacterial infection and auto-antigen stimulation. MALT lymphomas are derived from marginal-zone B cells in the Peyer's patch, which they still highly resemble. These cancerous cells proliferate and invade the epithelium, to form lymphoepithelial lesions (LEL). MALT lymphomas can remain localized or widely disseminate, and can also transform into diffuse large B cell lymphoma [33]. There is much evidence that gastric lymphoma arises from *H. pylori* associated acquired MALT in the gastric mucosa (where MALT is usually not found), one example being an association between *H. pylori* infection and the development of gastric lymphoma in a case controlled study [34]. Later experiments showed that *H. pylori* specific T cells are present in the tumour, and that they promote the growth of the B-cell tumour cells by CD40-mediated signalling [35]. Hence, the accepted view is that *H. pylori* induces accumulation of MALT, and then promotes chronic inflammation by activating a T cell response, which in turn causes DNA damage that eventually leads to the emergence of B cell neoplasia. On top of this, the B-cell lymphoma cells continue to be stimulated by auto-antigens directly, and by contact with *H. pylori* specific T cells. Indeed, antibiotic therapy in some cases leads to disease eradication [36].

#### 3.2.2. Viral mechanisms

As well as a link with bacterial infection, there is also a strong association between viral infection and lymphoma, including human T-cell lymphotropic virus-1 (HTLV-1), hepatitis C virus (HCV) and Epstein–Barr virus (EBV). HCV is the exception in that it is not thought to be a pure ‘oncogenic’ virus, but rather that it induces lymphoma by indirect means. Specifically, it is proposed that the E2 protein of HCV may cause chronic antigen-driven responses in B cells, leading to lymphomagenesis [37]. EBV and, to a lesser extent, HTLV-1 are oncogenic viruses in the traditional sense, though both generally also rely on normal mechanisms of mature lymphocyte proliferation to induce lymphoma. EBV is a ubiquitous virus that persistently infects greater than 90% of the worldwide population [38]. In certain geographical areas, such as Africa, where it is associated with Burkitt lymphoma, it is a major cause of disease, although it usually requires some form of immunosuppression in the individual, in particular due to other infections such as malaria, in order to drive proliferation of B cells [39]. However, in this latter case, tonic rather than active BCR signalling is considered the driver of lymphomagenesis whereby the PI-3 kinase pathway is triggered rather than the multitude of pathways including NFkB that are seen in active signalling [40]. Indeed, high rates of lymphoma have been observed in individuals that exhibit systemic immune suppression, whether that is due to autoimmune disease, organ transplantation or primary/acquired immunodeficiency, including HIV infection [41]. As immunodeficient individuals cannot eliminate infections effectively, yet retain B and T cells that can be activated, they are particularly prone to chronic antigenic stimulation of their B and T cells, which then promotes lymphomagenesis [42].

Of particular note are the lymphomas that arise in the context of autoimmune disorders, including Sjogren's syndrome (SS). SS is a rheumatic autoimmune disease characterized by lymphocytic infiltration and destruction of salivary and lacrimal glands [43]. The initiating factor may be viral: herpes virus 6, cytomegalovirus and EBV among others have been implicated [44], with persistent chronic stimulation being maintained by auto-antigens such as Ro/SSA and/or La/SSB. This drives the proliferation of specific B and T lymphocytes, increasing the likelihood of their transformation [45]. Thus, the major complication of this syndrome is lymphoma [46], with approximately 5% of SS patients developing lymphocyte malignancy, in particular MALT lymphoma [47]. Despite the fact that the development of B-cell lymphoma is more frequent, there are T-cell malignancies associated with this disease, with the most common sub-type being angioimmunoblastic T-cell lymphoma [48]. The number of different malignancies associated with SS suggest there is more than one ‘cell of origin’, but there is evidence that some of the B-cell lymphomas may initiate in CD27^+^ memory B cells attracted preferentially into inflamed salivary glands [49]. Other autoimmune disorders also increase the risk of developing lymphoma, including coeliac disease and dermatitis herpetiformis, which are both disorders triggered by gliadin. Coeliac disease in particular has been linked to the uncommon entropathy-type T-cell lymphoma of the small intestine [50].

#### 3.2.3. Self-antigen stimulation

As alluded to above, the source of the antigen is not necessarily derived from an external pathogen as it has also been shown to derive from self, whereby chronic self-antigen-induced signalling through the B-cell receptor (BCR) is implicated in lymphomagenesis. For example, in activated B-cell (ABC) DLBCL, chronic active BCR signalling is considered essential for the survival of these tumour cells and the mediators of this have been shown to range from self-antigens in apoptotic debris to self-glycoproteins [51,52]. In evidence, ABC DLBCL cell lines have prominent BCR clusters, characteristic of normal B cells that have been exposed to antigen [51]. Together, these data suggest a continual requirement for proximal BCR signalling in some lymphomas whether that be induced by an invading pathogen or self-antigen.

#### 3.2.4. Signalling through the T-cell receptor and lymphomagenesis

A role for signalling through the TCR and/or activation of signalling proteins proximal to this receptor has also been implicated in the pathogenesis of PTCL. For example, Itk-Syk and NPM-ALK fusion proteins have both been shown to mimic proximal TCR-induced signalling, in the former case by associating with lipid rafts via its pleckstrin homology (PH) domain and in the latter by indirect activation of AP1 and NFAT transcription factors [29,53–55]. In fact, Itk-Syk expression *in vivo* was shown to be a strong mimic of TCR signalling to the extent that it induced negative selection in the thymi of these mice [29]. Furthermore, activating mutations of the CD28 co-stimulatory receptor of T cells have been reported in a few cases of AITL and PTCL, NOS [56]. Whether exogenous antigen signalling through the TCR is also a driver for T-cell lymphomagenesis remains to be proven, but the fact that many human PTCL express gene signatures associated with activated T cells lends some support [57,58]. Indirect evidence is provided by the vav-Lin28b model in which expression of Lin28b led to a pro-inflammatory environment whereby tumour cells expressed NFkB and tumours contained many pro-inflammatory cytokines [30] and the association of ALCL with insect bites and breast implants [59–61]. In this vein, cutaneous T-cell lymphomas (CTCL) have long been associated with antigenic stimulus, most recently as a consequence of the bacterial product Staphylococcus enterotoxin A, albeit by indirect means [62]. This latter example alludes to a role for an inflammatory environment driving cellular proliferation which may enable the acquisition of further tumour-promoting mutations. This mechanism may account for many further mutations that have been described for PTCL in genes normally activated downstream of the antigen receptor/in response to a pro-inflammatory environment including for example STAT proteins [63,64].

In summary, the causes of the tumour-inducing chronic antigenic stimulation are varied, and lymphomagenesis initiated in mature lymphocytes often involves other mechanisms such as direct integration of oncogenic viral DNA, immunosuppression, mutation-inducing developmental processes (e.g. SHM and CSR) as well as exposure to environmental mutagens and undoubtedly others yet to be determined.

## 4. Mechanisms of lymphomagenesis in ‘immature’ lymphocytes

Compared with the wealth of evidence that lymphoma emerges from late stage lymphocytes, there has been to date less evidence that lymphomas begin from the opposite end of the developmental pathway. In part, this is due to the tendency of malignancies of progenitor B or T cells to produce lymphocytic leukaemia rather than lymphomas, with the major exception being LBL. LBL accounts for approximately 2% of all lymphomas, with the B-cell fraction accounting for less than 10% of those cases (the remainder being of immature T-cell lineage) [6]. Considering B-LBL specifically, the precursor origin of this disease is distinguished from mature B-cell neoplasms by the expression of markers of pro- or pre-B cells such as CD19 and terminal deoxytransferase (TdT), and by the absence of surface immunoglobulin [5].

Studies of mouse models have also shown how a precursor B cell can give rise to a malignancy. In one study, bone marrow B-cell progenitors that were isolated and infected with Myc-encoding retrovirus gave rise to B-cell lymphomas when reinjected into recipient mice if p53 was also inactivated in these cells [65]. The tumours resembled late pro-B/small pre-B cells and had not formed clonal rearrangements of the B-cell receptor. This suggests that loss of p53 and overexpression of myc is sufficient to cause lymphoma originating in early B-cell progenitors [65]. A more recent, related study also showed that B-cell progenitors may be more susceptible to c-Myc-induced lymphomagenesis than their mature counterparts. In this case, the cooperating mutation was the loss of Msh2, a key element of the DNA mismatch repair pathway [66].

The immunophenotype of T-LBL often matches early stages of T cell development in the thymus, with expression of proteins such as CD2, CD34 and CD1a in the absence of CD4 and CD8 [6]. It therefore follows that lymphomas resembling immature T cells originate in their immature thymic-resident counterparts whereby differentiation to a mature T-cell phenotype is inhibited. For example, conditional deletion of *Pten* in the thymus of mice causes peripheral lymphomas, yet the tumour cells still resemble immature thymic populations [67]. Therefore, T-LBL is considered to arise from immature lymphocytes and PTCL from mature circulating lymphocytes. Yet, given the plasticity of the haemopoietic system, one cannot rule out a thymic origin for peripheral disease if T-cell development is unaffected or even promoted by the driving oncogenic event.

## 5. An immature origin for a mature T-cell lymphoma?

As alluded to above, if tumour cells resembling immature thymocytes can present as a peripheral disease, can tumour cells representative of peripheral T cells also originate in thymic precursors but retain the ability to differentiate? This is probably dependent on the driving tumour-promoting event and subsequent contributory mutations, and whether they are able to drive and/or be permissive of a differentiation programme, while paradoxically retaining a stem cell programme or at a later point re-acquiring this ability (perhaps, as a consequence of differentiation into memory cells as indicated above or through activation via mutation of a stemness gene signature). In support, at least in mice, mature T cells appear to be resistant to transformation by oncogenes such as LMO2, TCL1 or ΔTrkA suggesting their expression must be targeted to memory T cells or other cells that already possess the ability to self-renew such as progenitor populations [68]. The challenge of identifying the cell of origin of a well characterized PTCL, ALCL, has led to discoveries that suggest this disease is indeed seeded in immature thymocytes and that these incipient tumour cells develop into a malignancy with a mature ‘activated-T-cell’ phenotype in at least a subset of cases.

### 5.1. Anaplastic large-cell lymphoma and attempts to model it *in vivo*

ALCL has an unusual presentation, often lacking in T cell-specific cell surface proteins, yet with features indicative of an activated mature T cell, making the natural progression of the disease difficult to discern. The first defining features of ALCL were discovered in the mid-1980s, when a series of diffuse large-cell lymphomas were shown to strongly express CD30, suggesting an activated lymphoid cell of origin [69]. This designation was further restricted to a T- or null-cell phenotype, due to the expression of T-cell-specific cell surface proteins such as CD4 and production of cytotoxic T-cell proteins like perforin [70], together with clonal TCR rearrangements [8], or in the latter case in the apparent absence of any lineage defining markers. Regardless, the presence of clonal TCR rearrangements in greater than 90% of all cases assigns a T-cell origin to this disease [70]. The disparities observed in cell surface marker expression profile have provided some controversy towards identifying the cell of origin for this disease; in some cases, CD25 is expressed in combination with CD4 indicating a regulatory T cell [71], whereas CD4 expression indicates a helper T cell and CD8 and/or perforin a cytotoxic cell, although expression of cytotoxic proteins has been linked to NPM-ALK activity [72]. Yet in some cases CD4 and perforin are expressed, and therefore it is not clear at which stage of T-cell development this cancer arose and which physiological processes may or may not contribute to the ensuing phenotype. Indeed, a gene expression profiling study of three distinct entities of ALCL, systemic ALK+, systemic ALK− and cutaneous ALK− could not assign the disease to CD4^+^, CD8^+^ or CD30^+^ T-cell origin [73].

What is clear is that a large majority of ALCL are associated with a specific chromosomal translocation, the t(2;5)(p23;q35), which was described in the early 1980s and was later identified to be the fusion of a kinase gene, *ALK*, to a nucleolar protein gene, *Nucleophosmin 1* (*NPM*) [23]. The translocation breakpoint gene product is the *NPM-ALK* fusion gene, which is oncogenic due to key structural features. The 5′ end of the fusion, NPM, has an oligomerization domain that causes the NPM-ALK fusion to form homodimers allowing the 3′ end of the fusion, the ALK kinase, to transphosphorylate becoming constitutively activated [74]. However, it should be noted that NPM-ALK transcripts are detected in cells of healthy individuals with a relatively high frequency, suggesting the fusion product alone is insufficient to induce ALCL [75].

There have been several efforts to model NPM-ALK driven ALCL *in vivo*, with the goal in part to define the cell of origin of the disease. The first attempts involved retroviral transduction of bone marrow *ex vivo* with NPM-ALK and then injecting the cells into irradiated mice, which produced immunoblast-like B cell lymphomas in peripheral nodes [76]. While this was the first confirmation that NPM-ALK has the ability to transform lymphocytes as a consequence of its constitutively active tyrosine kinase, it also suggests that it cannot drive a T-cell developmental pathway, although the bone marrow culture conditions employed in this study do favour B-cell development [76]. Subsequently, a number of models were designed to regulate NPM-ALK expression with promoters that are specific to particular haemopoietic lineages [77]. For example, the Vav promoter is known to drive expression in all lineages of the haemopoietic system including early progenitors [78], whereas the Lck proximal [79] and CD2 promoters [80] drive expression from early stages of T-cell development (DN2 and DN3, respectively). In an effort to effectively ‘screen’ the haematopoietic compartment for the preferential target of NPM-ALK activity, Turner *et al.* expressed NPM-ALK from the Vav promoter in transgenic mice [81]. However, Vav/NPM-ALK transgenic mice mainly developed plasmacytomas, suggesting that perhaps NPM-ALK is more potent when expressed in B cells or that, as expected and observed, B cells are more prone to transformation than T cells (B-cell lymphoma is far more prominent than T-cell lymphoma in humans). However, NPM-ALK-induced B-cell lymphoma does exist, although extremely rare [82]. These data suggest together that for mice to develop a T-cell lymphoma, targeted expression of NPM-ALK to thymocytes and/or mature T cells is required. However, this has also led to mixed results, with CD2 promoter-driven NPM-ALK transgenic mice also producing lymphomas that appear to be of a B-cell phenotype [83], and the CD4 promoter used in the same context leading in some cases to plasmacytomas [84], suggesting that either the promoters are ‘leaky’ in the transgenic setting or NPM-ALK can alter cell-lineage differentiation. Of course, when generating transgenic lines, one also has to consider the effects of site of integration of the transgene in this regard. Indeed, the CD4/NPM-ALK approach also gave rise to two other transgenic lines, one that produced plasmacytomas or thymic tumours, but another that exclusively develops thymic T-cell lymphomas [84]; the reasons for these disparate phenotypes from the same round of microinjection are not immediately apparent and might reflect previously discussed concepts. In a complementary approach, Lck promoter-driven NPM-ALK gave rise again to immature thymic T-cell tumours, but the disease was so aggressive the line was not able to be maintained [85]. Regardless, in no cases has a PTCL presented in mice expressing NPM-ALK in a transgenic context even though the CD4 promoter also drives expression in mature T cells (the CD4 promoter construct used to generate these mice lacks silencing elements that would normally supress expression in early thymocytes [86]). The consequences of driving NPM-ALK expression exclusively in mature T cells are yet to be explored, largely due to the current lack of any peripheral T-cell-specific promoters for use in the transgenic context (reviewed in [87]).

### 5.2. Evidence for anaplastic large cell lymphoma arising in progenitor T cells in the thymus

The difficulties in reproducing an ALCL-like disease in mice suggests that the oncogenic activity of NPM-ALK needs to be targeted to a specific stage of T-cell development, perhaps with one or more cooperating mechanisms. The resemblance of ALCL to an activated mature T cell, due to the expression of activated T-cell proteins such as CD30, granzyme B and perforin [88], has led most work to concentrate on mature subsets as the ‘normal counterpart’ of ALCL, with suggestions including IL-17-producing Th17 cells [89] or Foxp3 expressing T regs [71,90]. It has also been shown that mature human CD4^+^ T cells can be transformed by NPM-ALK *in vitro* [91]. An alternative hypothesis is that thymic expression of NPM-ALK is reflective of the human scenario but that other mechanism(s) contribute to the pathogenesis of the disease to eventually give rise to a peripheral lymphoma of apparent mature T cells. In support of this, transcripts for NPM-ALK have been detected in haematopoietic stem cell-rich cord blood of 1.95% of healthy newborns (*n* = 103) [92]. While this statistic does not concur with the low ALCL incidence of 1.2 million of the general population, it does suggest an early event is possible; indeed, the *NPM* promoter that drives expression of NPM-ALK in humans is active throughout thymic development [86]. Furthermore, ALCL, ALK+ is largely diagnosed in children and young adults (when the thymus is at its largest) and an *in utero* origin for the translocation is in fitting with previous studies of childhood leukaemia [93,94]. It therefore follows that if NPM-ALK expression in cord blood is the source of incipient tumour cells, then why is NPM-ALK expression largely restricted to PTCL in humans? The obvious answer to this is that secondary, perhaps peripheral events are important to disease pathogenesis although mediastinal involvement is observed in up to 50% of ALCL cases [95]. However, in support of a thymic presence of incipient tumour cells, our own studies turned towards human ALCL tumours and investigations for any clues that would point towards aberrant thymic events that might be induced by, and be a marker of the presence and expression of NPM-ALK in patient thymi [86]. Given that rearrangement of TCR genes is a tightly controlled and regulated process occurring in a temporal, step-wise manner and pertubations to this could indicate obstructive or at least subverted processes in the thymus. Indeed, our studies of CD4/NPM-ALK transgenic mice show a delay at the DN3 stage of thymic development, the point in thymic development at which β-selection is occurring (figure 2); it is therefore likely that if NPM-ALK is subverting processes in the human thymus, it would be at this stage when TCRβ chain rearrangement is taking place. This led us to assess by PCR and array CGH the clonality of TCR rearrangements of human ALK+ ALCL malignancies and expression of any consequent TCR. Around two-thirds of the samples tested had undergone major in-frame clonal TCRα rearrangement in the absence of a comparable major TCRβ clonal rearrangement [86]. There were minor clonal TCRβ rearrangements, thought to either be indicative of infiltrating inflammatory T cells or ongoing rearrangements within sub-clones of the major tumour cell clone. This is a TCR profile that is not consistent with normal thymocyte development but is consistent with observations made by ourselves and others that ALCL very rarely express the TCRβ protein by immunohistochemistry [86,96] and, more specifically, with our observation that NPM-ALK can replace the TCRβ signalling cascade in mice (CD4/NPM-ALK/RAG^−/−^ mice produce thymic lymphomas that appear to be mature, post TCR-rearrangement thymocytes) [53,97]. In further evidence of a thymic origin, studies of tumour-propagating cells in ALCL cell lines and patient tumours show an early thymic progenitor genetic signature [98]. Whether the thymic environment plays an essential role in tumorigenesis or if the aberrant thymic events detected are purely consequential of NPM-ALK-induced Notch 1 remains to be determined [86]. This then leaves the question of how primitive thymic T cells expressing NPM-ALK become mature T-cell lymphomas, especially given that expression of NPM-ALK in the thymi of mice fails to produce this disease phenotype [84].

### 5.3. Thymic nucleophosmin-anaplastic lymphoma kinase expression in the presence of a transgenic clonal T-cell receptor gives rise to peripheral T-cell lymphoma in mice

If ALCL originates in the thymus, then the cells must retain the ability to exit into the peripheral circulation providing they can bypass the above-discussed developmental stages. Thymic emigration is mediated by the sphingosine-1-phosphate receptor 1 (S1PR1), which is expressed on thymocytes and responds to an S1P gradient (reviewed in [99]), and while its expression has not been examined in ALCL or in the mouse models, it is possible that the presence of a transgenic TCR (as discussed below) might modulate its expression. S1PR1 is a G-protein-coupled receptor whose expression on SP CD4 or CD8 thymocytes is modulated by the strength of signal induced via the TCR on ligation with self-antigen presented in the context of MHC [99]. High-avidity interactions lead to akt-induced phosphorylation of FOXO1 preventing its activity as a transcription factor for S1PR1, thereby, preventing thymic export [99]. Given that NPM-ALK is able to mimic TCR proximal signalling, its co-expression with a transgenic TCR may alter presumed avidity for peptide-MHC and subsequent regulation of thymic export. Indeed, in the absence of examining the thymi of patients affected by ALCL, it was shown that backcrossing CD4/NPM-ALK to TCR transgenic mice (OTI; MHC class I restricted) led to PTCL development exactly mimicking the human disease [86]. Whether the observed phenotype is due to the combined effect of the transgenic TCR and NPM-ALK in activating proximal TCR signalling remains to be determined, but given the known role for TCR avidity and subsequent strength of the transduced signal in mediating thymic survival and emigration of T cells, this remains a distinct possibility. Furthermore, this peripheral phenotype was only observed on a RAG-competent background, suggesting that clonal competition with T cells expressing endogenously rearranged TCR might be a contributing factor; only germline endogenous TCR genes were present in tumours, suggesting either suppression of RAG-induced endogenous rearrangement in the presence of the TCR transgene or that T cells with endogenously rearranged transgenes are inhibited from tumour development [86]. Indeed, RAG-negative littermates, which would only produce clonal TCR transgenic receptor expressing T cells, did not develop lymphoid malignancy. This is in contrast with a previous study whereby NPM-ALK expression in transgenic TCR-expressing T cells only produced tumours in the absence of clonal competition (i.e. in the absence of polyclonal TCR-expressing T cells) [68].

These observations are in line with a study by Wang *et al.*, in which snf5 was conditionally deleted by the cre-loxP system with cre recombinase regulated by the CD2 promoter, which is expressed before progenitor cells enter the thymus [25]. In the CD2cre snf5^fl/fl^ mouse, T-cell development was blocked at the DN3 stage and no lymphoma developed, but introduction of the OT1 TCR transgene into the CD2cre snf5^fl/fl^ mouse resulted in the presence of mature T cells in the periphery and all mice developed CD3^+^CD8^+^ T-cell lymphomas. As previously mentioned, there is evidence of a DN3 block in the CD4/NPM-ALK mouse, suggesting similar mechanisms are at work in the CD2cre snf5^fl/fl^ model of lymphomagenesis and the CD4/NPM-ALK model. The difference appears to be that NPM-ALK-expressing cells are able to overcome the DN3 block, as seen in the CD4/NPM-ALK/RAG^−/−^ line, whereas snf5 loss is unable to do the same. However, the similarity is that the presence of the OT1 TCR was able to radically alter the phenotype of the model, and notably in neither study did the OT1 TCR need to be stimulated by ovalbumin administration to achieve this result [25,86]. This suggests that TCR signalling at the thymocyte stage rather than activation in the periphery is a crucial addition, perhaps simply as a way of overcoming the DN3 block in a manner that enables survival and emigration from the thymus.

### 5.4. Anaplastic large-cell lymphoma development and the role of the antigen receptor

As discussed above, the strength of signal transduced through the TCR combined with its modulation by NPM-ALK in the thymus might be critical for peripheral disease development. But what is clear is that in the established tumours, the TCR is often expressed in neither the NPM-ALK/OTI mouse model nor in human tumours, which would seem to preclude a role for antigen receptor signalling in the periphery in the progression of the disease. Supporting this supposition is the fact that ALCLs lack expression of TCR molecules or molecules of proximal TCR signalling [96], which might provide a selective advantage to the tumour cells as loss of such proteins has been implicated in protection from activation-induced cell death (AICD) in other lymphomas [100]. Despite this there are reasons to believe that TCR signalling does play an oncogenic role in the pathogenesis of ALCL: not only are TCRβ chain rearrangements found in the majority of cases, but more significantly the cells express proteins associated with activated T cells (CD30, perforin and Granzyme B) [70]. This suggests either that signalling via the TCR happened at some point prior to downregulation (the latter putatively via epigenetic mechanisms [101]), or alternatively NPM-ALK induces aberrant expression of these molecules. To support the latter point, it has been shown that NPM-ALK induces expression of cytotoxic molecules in ALCL [72]. However, it is also thought that a so-called ‘second hit’ of a foreign body triggering a strong immune response is crucial to the pathogenesis of ALCL. Indeed, there is a strong association of systemic ALK-positive ALCL presenting with skin lesions occurring after an insect bite [59]. The hypothesis is that the release of cytokines at the site of the bite elicits the activation and proliferation of T cells bearing the NPM-ALK translocation. As such, one cannot preclude an innate lymphoid cell-type reaction of the tumour cells to inflammation, independent of a TCR. Regardless, complementary to investigating the potential cell of origin, the role of TCR signalling in ALCL is pertinent in order to determine the extent to which T-cell activation is relevant to the generation of the disease either as an oncogene or tumour suppressor that must be silenced.

Supporting the latter, exposure of the CD4/NPM-ALK/OT1 mouse to MHV-OVA abrogated ALCL development in favour of hepatocellular carcinomas and sarcoma, as seen in the CD4/NPM-ALK/RAG^−/−^/OT1 mice [86]. This demonstrates that cognate peripheral TCR signalling in NPM-ALK-expressing cells is not compatible with ALCL development and/or survival. Thus, coexistence with a functional TCR allows thymic egress, but also subsequent cell death in the presence of simultaneous TCR and NPM-ALK signalling, by a process analogous to thymic negative selection. As such, the TCR acts as a tumour suppressor, which must be downregulated for development of classical, peripheral ALCL. Indeed, it has been shown that epigenetic mechanisms are induced by NPM-ALK to downregulate proteins of proximal TCR signalling [101,102]. This goes some way to explain the apparent contradictions in the commonly rearranged but not expressed TCR in ALCL.

## 6. Conclusion

**Figure 3.**
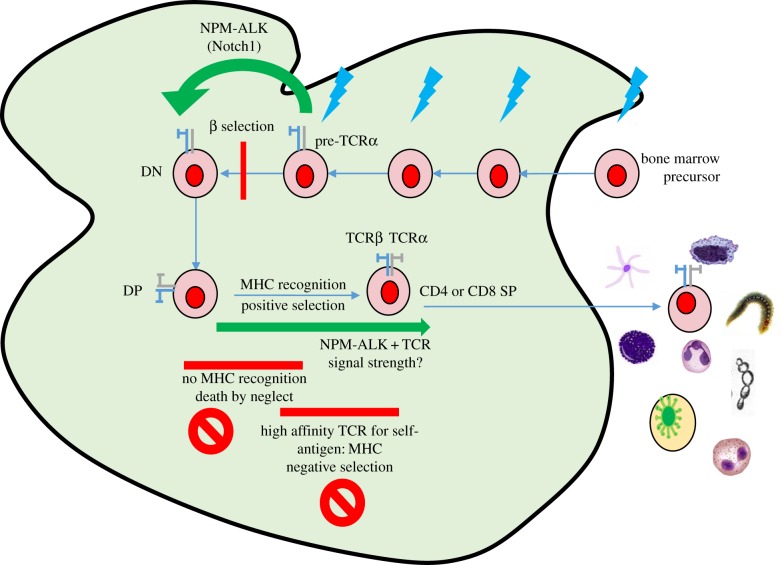
Mechanisms of ALCL pathogenesis. The generation of the t(2;5), variant translocation or other oncogenic driver event occurs in a T-cell progenitor (blue arrows) and is permissive of TCR VDJ recombination but also facilitates bypass of the beta selection checkpoint, enabling cells with TCR that would normally lead to cell death to survive. These cells must then be positively selected, suggesting that either low-affinity TCR are expressed and/or that NPM-ALK mimics a TCR signal of correct intensity permissive of survival. NPM-ALK-expressing T cells emigrate into the periphery and, dependent on the inflammatory microenvironment encountered and bodily location, differentiate into distinct T-cell subsets potentially in a TCR-independent manner (akin to innate lymphoid cells) or following selection of cells that have downregulated the TCR (or proteins involved in proximal TCR signalling). Owing to the acquisition of further (epi)genetic events, the transformed cells clonally expand independently of the source of inflammation. DN, double negative; DP, double positive; SP, single positive.

For most in the field, the consensus has been that B- and T-cell lymphomas emerge in developmentally late-stage, differentiated lymphocytes, with chronic antigen stimulation being a key initiator and driver of disease. This is for good reason as there are numerous examples to support this. However, it is possible that the ‘seeds’ of these mature diseases are sown much earlier in development, with predisposing genetic events occurring in primitive cells and the final disease phenotype shaped by activities occurring in the developing differentiated cell. Notably for ALCL, a lymphoma that would appear a prime candidate for an origin in an ‘activated’ mature cytotoxic T cell, new evidence suggests that a thymic origin is a possibility in a portion of the cases (figure 3). The importance of this distinction is that while lymphomas are generally defined by their appearance and location in the periphery, effective treatment and relapse prevention requires eradication of all pools of transformed cells. Thus, by showing that progenitor lymphocytes can be the origin for T-cell lymphomas such as ALCL, we ensure that we can eliminate any reservoir that could re-establish the disease.
